# Reference Values and Age Differences in Body Composition of Community-Dwelling Older Japanese Men and Women: A Pooled Analysis of Four Cohort Studies

**DOI:** 10.1371/journal.pone.0131975

**Published:** 2015-07-06

**Authors:** Satoshi Seino, Shoji Shinkai, Katsuya Iijima, Shuichi Obuchi, Yoshinori Fujiwara, Hideyo Yoshida, Hisashi Kawai, Mariko Nishi, Hiroshi Murayama, Yu Taniguchi, Hidenori Amano, Ryutaro Takahashi

**Affiliations:** 1 Tokyo Metropolitan Institute of Gerontology, 35–2 Sakae, Itabashi, Tokyo, Japan; 2 Institute OF Gerontology, The University of Tokyo, 7-3-1 Hongo, Bunkyo, Tokyo, Japan; University of Leipzig, GERMANY

## Abstract

**Objectives:**

To determine age- and sex-specific body composition reference values and investigate age differences in these parameters for community-dwelling older Japanese men and women, using direct segmental multi-frequency bioelectrical impedance analysis.

**Methods:**

We conducted a pooled analysis of data collected in four cohort studies between 2008 and 2012: Kusatsu Longitudinal Study, Hatoyama Cohort Study, Itabashi Cohort Study, and Kashiwa Cohort Study. The pooled analysis included cross-sectional data from 4478 nondisabled, community-dwelling adults aged 65-94 years (2145 men, 2333 women; mean age: 72.9 years in men and 72.6 years in women). Body weight, fat mass (FM), percentage FM, fat-free mass (FFM), and appendicular lean soft tissue mass were measured using the InBody 720 and 430 (Biospace Co. Ltd, Seoul, Korea). The values were then normalized by height in meters squared to determine body mass index (BMI), FM index (FMI), FFM index (FFMI), and skeletal muscle mass index (SMI).

**Results:**

Simple means (standard deviation) of BMI, percentage FM, FMI, FFMI, and SMI were 23.4 (2.9) kg/m^2^, 24.9 (6.3)%, 5.96 (2.09) kg/m^2^, 17.4 (1.5) kg/m^2^, and 7.29 (0.76) kg/m^2^, respectively, in men and 22.7 (3.3) kg/m^2^, 31.7 (7.1)%, 7.40 (2.61) kg/m^2^, 15.3 (1.2) kg/m^2^, and 5.86 (0.67) kg/m^2^, respectively, in women. We then calculated quartiles and quintiles for these indices after stratifying for sex and 5-year age group. FFMI and SMI decreased significantly with age in both sexes (*P* < 0.001 for trends), but FFMI remained constant among the women with only a 1% decrease up to age 84 years. Percentage FM increased significantly, with age (*P* < 0.001 in men and *P* = 0.045 in women for trends), but FMI was unchanged in both sexes (*P* = 0.147 in men and *P* = 0.176 in women for trends).

**Conclusion:**

The present data should be useful in the clinical evaluation of body composition of older Japanese and for international comparisons. The small age-related decrease in FFMI may be a noteworthy characteristic of body composition change in older Japanese women.

## Introduction

In general, fat-free mass (FFM), which includes lean soft tissue mass (LM) and bone mineral content, decreases gradually during old age, along with an increase in fat mass (FM) [1]. Although body mass index (BMI) is widely used as a simple surrogate indicator of body composition, measurement of BMI alone may mask age-related changes in FFM and FM that occur in later life. VanItallie et al [2]. suggested partitioning BMI into an FFM index (FFMI) and an FM index (FMI), analogous to BMI, that could help track body composition and nutritional status. Han et al [3]. reported that FFMI (which they described as lean mass index) was a better predictor than BMI of mortality among older Korean adults. Loss of FFM is the key component of sarcopenia; loss of skeletal muscle mass, strength, and function is what leads to functional disability [4]. Therefore, focusing attention on not only BMI, but also FFM, FM, and skeletal muscle mass, is particularly important in aging studies.

Dual-energy X-ray absorptiometry (DXA) is the generally accepted method for directly assessing total and regional FM, LM, and bone mineral density [5]. However, it is not widely available outside clinical and research settings due to lack of access to and the high cost of the equipment, risk of radiation exposure, and required training. In recent years, advances in bioelectrical impedance analysis (BIA) devices have greatly increased acceptance of BIA in assessing body composition in research and clinical settings. The Asian Working Group for Sarcopenia (AWGS) [4] recommended BIA as the main approach for sarcopenia assessment in community-based screening, because of its portability, reasonable cost as compared with other methods, noninvasiveness, radiation-free functioning, and ease of use. Specifically, direct segmental multi-frequency BIA (DSM-BIA) has been shown to be more accurate than conventional BIA devices [6]. Although validation studies of the DSM-BIA using DXA as a reference standard have yielded mixed results [7–12], DSM-BIA was reported to have acceptable accuracy for estimating whole-body and appendicular FM and LM in validation studies among older Japanese [10, 12]. Therefore, DSM-BIA is useful for measuring the body composition of older Japanese in large-population research settings.

Several studies reported body composition reference values obtained by DXA and single-frequency BIA [13–17]; however, no published study presented age- and sex-specific comprehensive reference values for BMI, FFMI, FMI, and skeletal muscle mass index (SMI) [18] as measured by DSM-BIA among older Japanese adults. Because the characteristics of body composition vary by population [19, 20], it is important to present population-specific body composition reference values and discuss differences between populations. Therefore, this study aimed to establish age- and sex-specific body composition reference values for older Japanese adults, using large scale pooled data from four cohort studies. In addition, we investigated cross-sectional, age-related changes in these parameters.

## Materials and Methods

### Ethics Statements

All studies included in the pooled analysis were conducted with the approval of the Ethical Committee of the Tokyo Metropolitan Institute of Gerontology or Graduate School of Medicine, The University of Tokyo. All participants provided written informed consent.

### Data sources and study population

**Fig 1:**
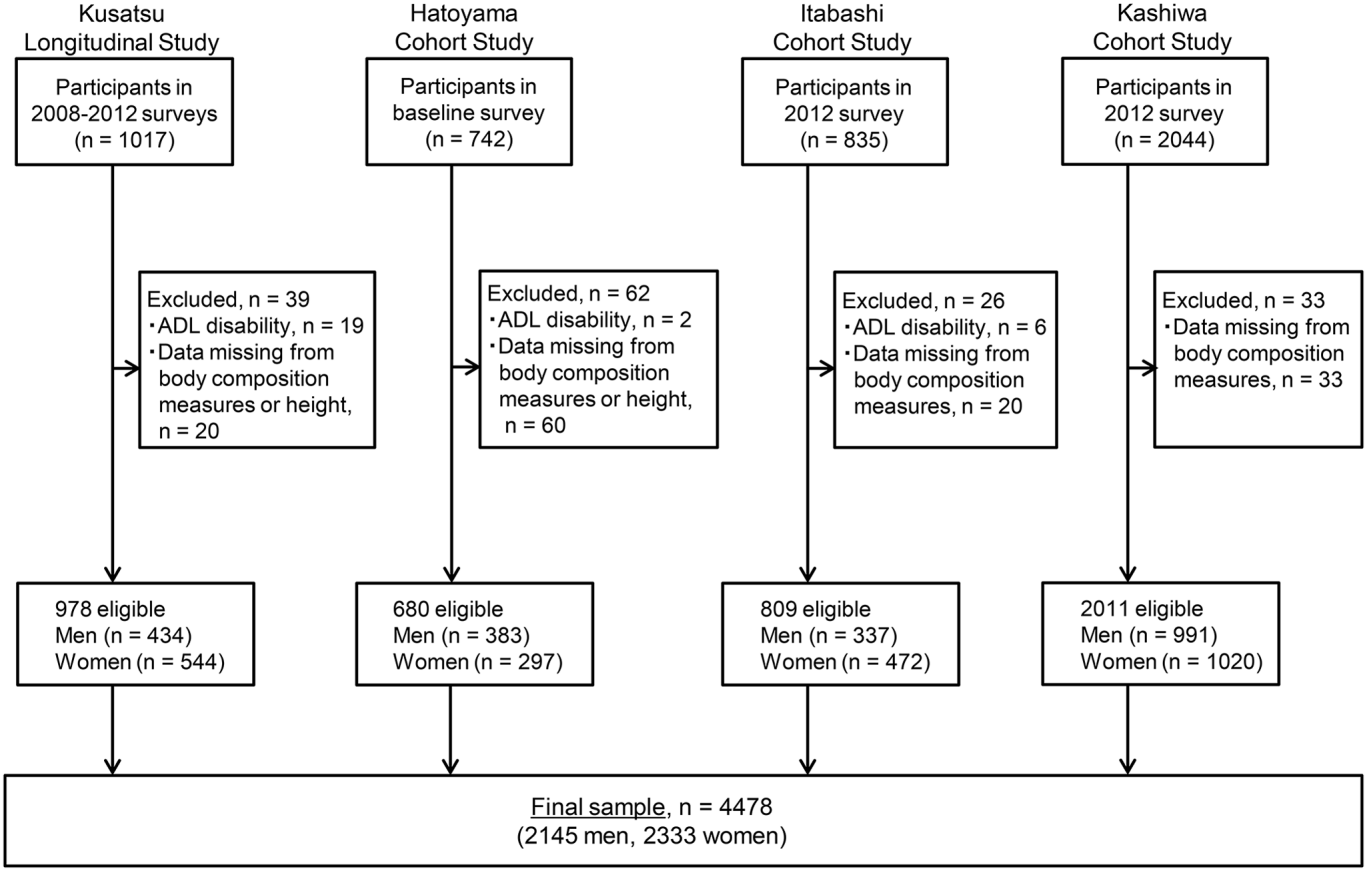
Schematic of participant selection processes in each included cohort study. ADL = activities of daily living.

The present study analyzed data from four cohort studies: the Kusatsu Longitudinal Study (KUSATSU) [21], Hatoyama Cohort Study (HATOYAMA) [22], Itabashi Cohort Study (ITABASHI) [23], and Kashiwa Cohort Study (KASHIWA) [24]. We used baseline data or data from the year when DSM-BIA was used to measure body composition parameters. The details of participant selection for the present study are shown in Fig 1.

### Kusatsu Longitudinal Study (KUSATSU)

The KUSATSU study is a longitudinal study on aging and health of older adults living in Kusatsu town, a rural community in northwest Gunma Prefecture, Japan [21]. The target population comprised National Health Insurance beneficiaries aged 65–74 years and individuals 75 years or older in the Medical Insurance System for the Elderly Aged 75 or Over. We used data when the subject first participated in health checkups between 2008 and 2012. The annual participation rate in health checkups was approximately 30% of the target population. A total of 1017 people (449 men and 568 women) were included in the study.

### Hatoyama Cohort Study (HATOYAMA)

The HATOYAMA study is a prospective cohort study of community-dwelling people aged 65 years or older living in the town of Hatoyama in Saitama Prefecture, Japan. The details of the participant selection process were previously described [22]. Briefly, 2697 residents (1354 men, 1343 women) aged 65–84 years were originally selected using stratified sampling, classified by age and residential area, and random sampling strategies. Of those people, 751 participated in a baseline survey in 2010 (participation rate, 27.8%), but nine persons declined to participate in the study. A total of 742 people (428 men and 314 women) were included in the study.

### Itabashi Cohort Study 2011 (ITABASHI)

In the ITABASHI study, 7162 residents aged 65–84 years living in nine residential areas of the city were recruited in 2011. After excluding 463 people who were institutionalized or overlapped with previous studies, 6699 (3136 men, 3563 women) were invited to participate in health checkups. In October 2011, 913 ambulatory residents received health checkups at the survey (participation rate, 13.6%) [23]. We used data obtained at a 1-year follow-up survey conducted in 2012 because the study started using DSM-BIA from that year onward. A total of 835 people (350 men and 485 women) participated in the follow-up survey and were included in this study.

### Kashiwa Cohort Study (KASHIWA)

The KASHIWA study is a prospective cohort study designed to characterize the biological, psychosocial, and functional changes associated with aging in community-dwelling older adults [24]. Originally, a total of 12,000 community-dwelling, functionally independent (i.e., not requiring nursing care provided by long-term care insurance) adults aged 65 years or older were randomly recruited from the resident register of Kashiwa city, a commuter town for Tokyo, in Chiba prefecture, Japan. Of these adults, 2044 people (1013 men and 1031 women) received baseline examinations (participation rate, 17.0%). The sample reflected the age distribution of residents in Kashiwa city for each sex [24].

### Final sample size

Among the participants (n = 4638) in the pooled data of the present study, individuals were excluded if they were dependent in any of the five basic activities of daily living, (i.e., bathing, dressing, walking, eating, or continence) or if they had missing data for height or body composition (including participants with inappropriate values, such as negative values). The final, pooled sample size was 4478 (2145 men and 2333 women aged 65–94 years).

### Assessment of health-related information

Age, body height, and history of chronic disease (hypertension, stroke, heart disease, and diabetes mellitus) were assessed in all cohorts. History of chronic disease was confirmed in face-to-face interviews by physicians or nurses.

### Assessment of body composition measurements

The KUSATSU, HATOYAMA, and ITABASHI studies used the InBody 720 analyzer (Biospace Co. Ltd, Seoul, Korea) for the DSM-BIA [10, 12], and the KASHIWA study used the InBody 430 analyzer (Biospace Co. Ltd, Seoul, Korea) [24]. These body composition analyzers adopt a tetrapolar, eight-point tactile electrode system that separately measures impedance of the arms, trunk, and legs at six different frequencies (1, 5, 50, 250, 500, and 1000 kHz) in the InBody 720 or three different frequencies (5, 50, and 250 kHz) in the InBody 430, for each segment. We combined data from the InBody 720 and 430 because the use of three different frequencies (i.e., 5, 50, and 250 kHz) was sufficient to measure the body composition parameters used in this study [25].

Before measurement, participants wiped the bottom of their feet with an electrolyte tissue. Then, they were instructed to stand on the scale while holding the handrails with metal grip electrodes, thereby providing contact with a total of eight electrodes (two for each foot and hand). The participants fully extended their arms at an abduction angle of approximately 20 degrees laterally. The InBody 720 and 430 automatically estimate weight, BMI, FM, percentage FM, FFM, and LM of the arms and legs. We calculated appendicular LM (ALM) as the sum of the LM of the arms and legs. The FM, FFM, and ALM were then normalized by height in meters squared, to determine FMI [2], FFMI [2], and SMI [18]. The sum of FFMI and FMI is mathematically equivalent to BMI. We divided BMI into three categories (<18.5 kg/m^2^, 18.5–24.9 kg/m^2^, and ≥25.0 kg/m^2^) and defined a low SMI as a value <7.0 kg/m^2^ in men or <5.7 kg/m^2^ in women, according to the AWGS criteria [4]. Furthermore, we calculated ALM-to-BMI ratio (ALM_BMI_) [26].

### Statistical analyses

We used descriptive statistics to characterize the study population. We used the Kolmogorov-Smirnov *W* test to check data for normality and analyzed differences in characteristics between men and women using the unpaired *t* test and chi-square test. The means and standard deviations (SDs) of body composition parameters were tabulated per 5-year age group (65–69, 70–74, 75–79, 80–84, and 85 years or older) for each sex. For each body composition parameter in each 5-year age group, we calculated the percentage difference as compared with values from the age group 65–69 years. We also performed a random effects meta-analysis to test heterogeneity across studies for all age strata, using *Q* and *I* ^2^ statistics [27]. To evaluate linear trends in the means of body composition parameters between age groups, we used weighted one-way analyses of variance by sex. Finally, we calculated quartiles and quintiles (20^th^, 25^th^, 40^th^, 50^th^, 60^th^, 75^th^, and 80^th^ percentiles) of each body composition parameter after stratification for sex and 5-year age group.

We used an alpha level of 0.05 to identify statistical significance and performed all statistical analyses using IBM SPSS Statistics Version 20.

## Results

Table 1 summarizes the characteristics of the study population. There was no significant difference in age distribution between sexes, and the sample size was relatively small for the age group 85 years or older in both sexes. FM, percentage FM, and FMI were significantly higher in women than in men; however, FFM, FFMI, ALM, SMI and ALM_BMI_ were significantly higher in men than in women. On the basis of the AWGS criteria, 708 (33.0%) men and 930 (39.9%) women had a low SMI, and the prevalence of low SMI was significantly higher in women than in men. The descriptive details of the study participants by cohort are available in the Supporting Information (S1 Table [men] and S2 Table [women]).

**Table 1:** Characteristics of the study participants (n = 4478).

Variables	Mean ± SD or n (%)				*P* value
	Men (n = 2145)		Women (n = 2333)		
Age, years	72.9 ± 5.6		72.6 ± 5.6		0.086
Age group, n (%)					0.465
65–69	725	(33.8)	833	(35.7)	
70–74	646	(30.1)	697	(29.9)	
75–79	471	(22.0)	511	(21.9)	
80–84	236	(11.0)	232	(9.9)	
85^+^	67	(3.1)	60	(2.6)	
Geographic area, n (%)					< 0.001
KUSATSU	434	(20.2)	544	(23.3)	
HATOYAMA	383	(17.9)	297	(12.7)	
ITABASHI	337	(15.7)	472	(20.2)	
KASHIWA	991	(46.2)	1020	(43.7)	
Chronic disease, n (%)					
Hypertension (n = 4473)	1015	(47.4)	941	(40.4)	< 0.001
Stroke (n = 4473)	171	(8.0)	112	(4.8)	< 0.001
Heart disease (n = 4472)	430	(20.1)	281	(12.1)	< 0.001
Diabetes mellitus (n = 4471)	353	(16.5)	208	(8.9)	< 0.001
Height, cm	163.4 ± 6.0		150.4 ± 5.8		< 0.001
Weight, kg	62.5 ± 9.0		51.3 ± 8.0		< 0.001
BMI, kg/m^2^	23.4 ± 2.9		22.7 ± 3.3		< 0.001
BMI category					< 0.001
<18.5	90	(4.2)	196	(8.4)	
18.5–24.9	1481	(69.0)	1636	(70.1)	
> = 25.0	574	(26.8)	501	(21.5)	
FM, kg	15.9 ± 5.6		16.7 ± 5.8		< 0.001
Percentage FM, %	24.9 ± 6.3		31.7 ± 7.1		< 0.001
FMI, kg/m^2^	5.96 ± 2.09		7.40 ± 2.61		< 0.001
FFM, kg	46.6 ± 5.5		34.6 ± 3.8		< 0.001
FFMI, kg/m^2^	17.4 ± 1.5		15.3 ± 1.2		< 0.001
ALM, kg	19.5 ± 2.8		13.3 ± 2.1		< 0.001
SMI, kg/m^2^	7.29 ± 0.76		5.86 ± 0.67		< 0.001
Low SMI, n (%)*	708	(33.0)	930	(39.9)	< 0.001
ALM_BMI_	0.841 ± 0.119		0.594 ± 0.095		< 0.001

SD = standard deviation, KUSATSU = Kusatsu Longitudinal Study, HATOYAMA = Hatoyama Cohort Study, ITABASHI = Itabashi Cohort Study, KASHIWA = Kashiwa Cohort Study.

BMI = body mass index: weight (kg)/height (m)^2^, FM = fat mass, FMI = fat mass index: FM (kg)/height (m)^2^, FFM = fat-free mass, FFMI = fat-free mass index: FFM (kg)/height (m)^2^, ALM = appendicular lean soft tissue mass, SMI = skeletal muscle mass index: ALM (kg)/height (m)^2^. ALM_BMI_ = ALM-to-BMI ratio: ALM (kg)/BMI (kg/m^2^).

*Low SMI is defined as <7.0 kg/m^2^ in men or <5.7 kg/m^2^ in women, using the Asian Working Group for Sarcopenia criteria [4].

**Fig 2:**
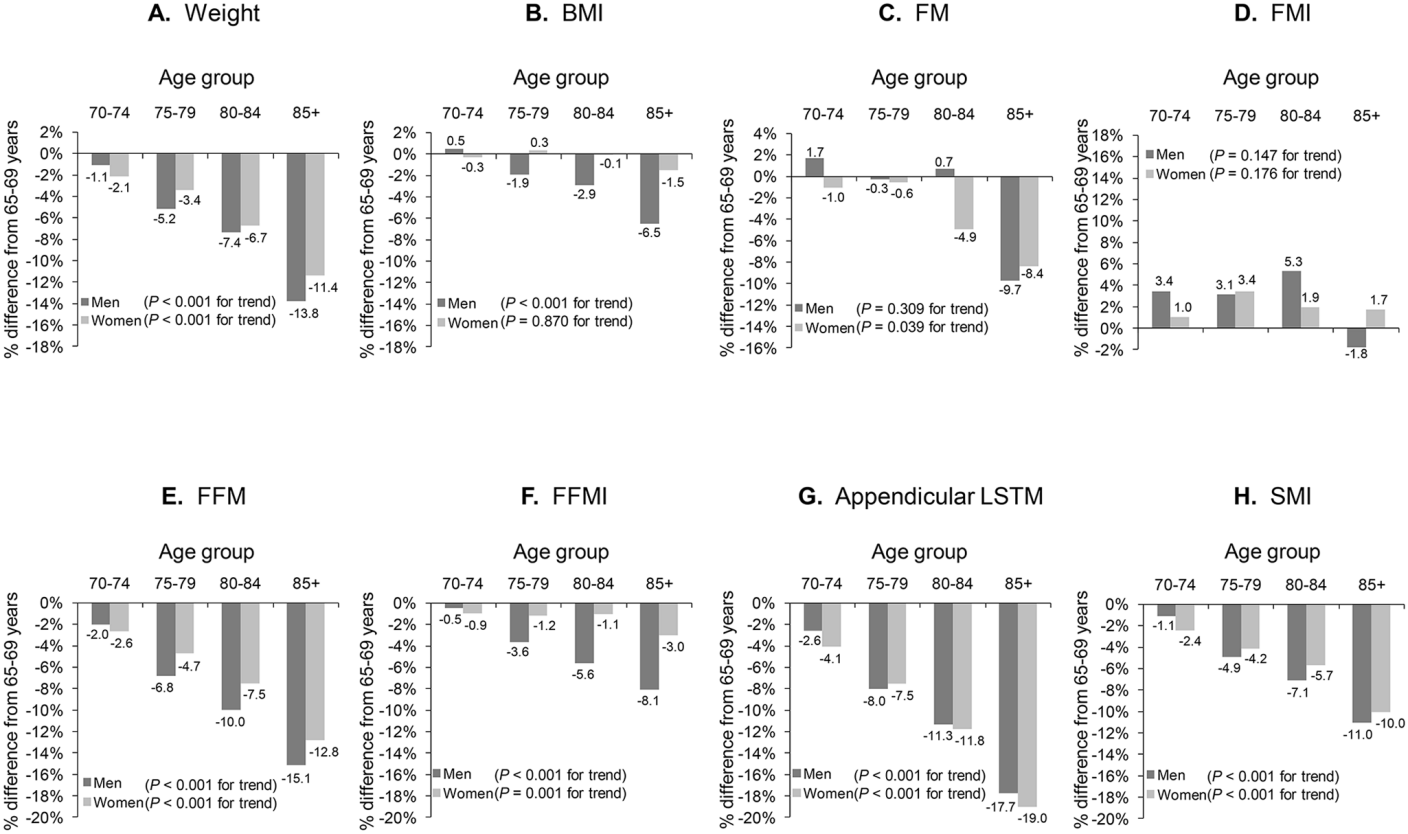
Percentage by which body composition parameters (A-H) in each age group and sex differed from those in age group 65–69 years. **A** = weight, **B** = body mass index (BMI): weight (kg)/height (m)^2^, **C** = fat mass (FM), **D** = fat mass index (FMI): FM (kg)/ height (m)^2^, **E** = fat-free mass (FFM), **F** = fat-free mass index (FFMI): FFM (kg)/ height (m)^2^, **G** = appendicular lean soft tissue mass (ALM), and **H** = skeletal muscle mass index (SMI): ALM (kg)/ height (m)^2^.

Tables 2 and 3 show the means and SDs for body composition parameters according to age group in men and women, respectively. Fig 2 shows the percentage differences in weight, FM, FFM, ALM and their indices for each age group as compared with respective values for the age group 65–69 years (which we refer to as age-related change in this study). In both sexes, weight, FFM, FFMI, ALM, and SMI showed significant decreasing trends with advancing age. Although the age-related changes in weight, FFM, and ALM diminished in both sexes when indexed, age-related decreases in BMI, FFMI, and SMI were still pronounced in men. BMI had a significant decreasing trend in men but was unchanged in women. In contrast, although absolute FM remained unchanged in men, there was a significant downward trend in women. Percentage FM showed significant increasing trends in both sexes, but FMI did not change in either sex.

**Table 2:** Descriptive statistics for body composition parameters, by age group (men).

	Mean ± standard deviation						
Variables		Age group					*P*
	Overall	65–69	70–74	75–79	80–84	85^+^	for trend
	(n = 2145)	(n = 725)	(n = 646)	(n = 471)	(n = 236)	(n = 67)	
Height, cm	163.4± 6.0	165.0± 5.8	163.7± 6.0	162.3± 5.5	161.1± 5.5	158.5± 6.3	< 0.001
Weight, kg	62.5 ± 9.0	64.2 ± 9.1	63.5 ± 8.5	60.9 ± 8.6	59.5 ± 9.1	55.4 ± 8.1	< 0.001
BMI, kg/m^2^	23.4 ± 2.9	23.6 ± 3.0	23.7 ± 2.8	23.1 ± 2.9	22.9 ± 3.1	22.0 ± 2.9	< 0.001
FM, kg	15.9 ± 5.6	15.9 ± 5.8	16.1 ± 5.3	15.8 ± 5.5	16.0 ± 5.8	14.3 ± 5.3	0.309
Percentage FM, %	24.9 ± 6.3	24.1 ± 6.3	24.9 ± 5.9	25.4 ± 6.3	26.2 ± 6.4	25.3 ± 6.9	< 0.001
FMI, kg/m^2^	5.96 ± 2.09	5.83 ± 2.15	6.03 ± 2.00	6.01 ± 2.06	6.14 ± 2.19	5.72 ± 2.15	0.147
FFM, kg	46.6 ± 5.5	48.4 ± 5.3	47.4 ± 5.3	45.1 ± 4.9	43.5 ± 4.8	41.0 ± 4.9	< 0.001
FFMI, kg/m^2^	17.4 ± 1.5	17.7 ± 1.4	17.7 ± 1.4	17.1 ± 1.4	16.7 ± 1.4	16.3 ± 1.4	< 0.001
ALM, kg	19.5 ± 2.8	20.4 ± 2.6	19.9 ± 2.7	18.8 ± 2.6	18.1 ± 3.0	16.8 ± 2.7	< 0.001
SMI, kg/m^2^	7.29 ± 0.76	7.48 ± 0.67	7.40 ± 0.72	7.11 ± 0.75	6.95 ± 0.89	6.66 ± 0.77	< 0.001
ALM_BMI_	0.841 ± 0.119	0.873 ± 1.090	0.846 ± 0.116	0.819 ± 0.112	0.797 ± 0.133	0.770 ± 0.126	< 0.001

BMI = body mass index: weight (kg)/height (m)^2^, FM = fat mass, FMI = fat mass index: FM (kg)/height (m)^2^, FFM = fat-free mass, FFMI = fat-free mass index: FFM (kg)/height (m)^2^, ALM = appendicular lean soft tissue mass, SMI = skeletal muscle mass index: ALM (kg)/height (m)^2^. ALM_BMI_ = ALM-to-BMI ratio: ALM (kg)/BMI (kg/m^2^).

**Table 3:** Descriptive statistics for body composition parameters, by age group (women).

	Mean ± standard deviation						
Variables		Age group					*P*
	Overall	65–69	70–74	75–79	80–84	85^+^	for trend
	(n = 2333)	(n = 833)	(n = 697)	(n = 511)	(n = 232)	(n = 60)	
Height, cm	150.4± 5.8	152.1± 5.0	150.8± 5.7	149.4± 5.6	147.1± 6.3	144.2± 6.2	< 0.001
Weight, kg	51.3 ± 8.0	52.5 ± 7.9	51.4 ± 7.9	50.8 ± 7.8	49.0 ± 7.4	46.6 ± 8.3	< 0.001
BMI, kg/m^2^	22.7 ± 3.3	22.7 ± 3.2	22.6 ± 3.4	22.8 ± 3.4	22.7 ± 3.1	22.4 ± 3.6	0.870
FM, kg	16.7 ± 5.8	16.9 ± 5.8	16.7 ± 5.7	16.8 ± 5.9	16.0 ± 5.7	15.5 ± 6.0	0.039
Percentage FM, %	31.7 ± 7.1	31.4 ± 6.9	31.7 ± 6.8	32.2 ± 7.3	31.9 ± 7.7	32.2 ± 7.7	0.045
FMI, kg/m^2^	7.40 ± 2.61	7.30 ± 2.52	7.37 ± 2.59	7.55 ± 2.75	7.44 ± 2.64	7.43 ± 2.80	0.176
FFM, kg	34.6 ± 3.8	35.7 ± 3.6	34.7 ± 3.7	34.0 ± 3.6	33.0 ± 3.6	31.1 ± 3.5	< 0.001
FFMI, kg/m^2^	15.3 ± 1.2	15.4 ± 1.2	15.3 ± 1.2	15.2 ± 1.2	15.2 ± 1.2	14.9 ± 1.3	0.001
ALM, kg	13.3 ± 2.1	13.9 ± 1.9	13.4 ± 2.0	12.9 ± 2.0	12.3 ± 2.0	11.3 ± 2.0	< 0.001
SMI, kg/m^2^	5.86 ± 0.67	6.01 ± 0.61	5.86 ± 0.66	5.76 ± 0.69	5.67 ± 0.69	5.41 ± 0.75	< 0.001
ALM_BMI_	0.594 ± 0.095	0.621 ± 0.087	0.598 ± 0.093	0.574 ± 0.093	0.550 ± 0.099	0.510 ± 0.084	< 0.001

BMI = body mass index: weight (kg)/height (m)^2^, FM = fat mass, FMI = fat mass index: FM (kg)/height (m)^2^, FFM = fat-free mass, FFMI = fat-free mass index: FFM (kg)/height (m)^2^, ALM = appendicular lean soft tissue mass, SMI = skeletal muscle mass index: ALM (kg)/height (m)^2^. ALM_BMI_ = ALM-to-BMI ratio: ALM (kg)/BMI (kg/m^2^).

The weighted means of body composition parameters calculated by a random effects meta-analysis are shown in the Supporting Information (S3 Table). The weighted means from a random effects meta-analysis model were very similar to unweighted simple means for body composition parameters. The *Q* statistics for all age strata had probability levels exceeding 0.05 (*I* ^2^ = 0.0–35.3% in men and 0.0–26.4% in women), indicating that the studies were homogeneous within strata in both sexes (S3 Table).

The prevalence of low SMI, as defined by AWGS criteria, by age group (i.e., 65–69, 70–74, 75–79, 80–84, and 85 years or older) was 155 (21.4%), 168 (26.0%), 206 (43.7%), 130 (55.1%), and 49 (73.1%), respectively, in men and 249 (29.9%), 280 (40.2%), 230 (45.0%), 129 (55.6%), and 42 (70.0%), respectively, in women.

Finally, Tables 4 and 5 show quartiles and quintiles of BMI, percentage FM, FMI, FFMI, SMI, and ALM_BMI_ by age group in men and women, respectively. The quartiles and quintiles of absolute variables (height, weight, FM, FFM, and ALM) are included in the Supporting Information (S4 Table).

**Table 4:** Quartiles and quintiles of body composition indices, by age group (men).

Body composition	Percentiles	Age group					
parameters		Overall	65–69	70–74	75–79	80–84	85^+^
BMI, kg/m^2^	80th	25.6	25.7	25.9	25.4	25.3	25.2
	75th	25.1	25.2	25.3	25.0	24.4	24.4
	60th	24.0	24.0	24.3	23.9	23.3	22.8
	50th	23.3	23.4	23.6	23.2	22.5	21.8
	40th	22.6	22.7	22.9	22.4	22.0	21.0
	25th	21.5	21.6	21.9	21.3	21.2	19.9
	20th	21.1	21.2	21.4	20.7	20.7	19.3
Percentage FM, %	80th	30.1	28.9	29.9	30.9	32.0	32.9
	75th	28.9	27.9	28.7	30.0	30.4	30.7
	60th	26.4	25.3	26.4	27.5	27.8	27.3
	50th	24.8	23.9	24.8	25.9	26.1	25.0
	40th	23.4	22.7	23.3	24.1	24.6	23.7
	25th	20.8	20.2	21.5	21.1	22.0	19.9
	20th	19.7	19.1	20.1	19.9	20.9	17.9
FMI, kg/m^2^	80th	7.63	7.37	7.71	7.77	7.78	7.73
	75th	7.23	6.96	7.30	7.40	7.47	7.24
	60th	6.29	6.01	6.29	6.50	6.42	6.25
	50th	5.78	5.60	5.87	5.98	5.83	5.58
	40th	5.33	5.12	5.41	5.49	5.44	5.04
	25th	4.49	4.35	4.71	4.49	4.67	4.21
	20th	4.23	4.12	4.36	4.23	4.31	3.57
FFMI, kg/m^2^	80th	18.6	18.8	18.8	18.3	17.9	17.5
	75th	18.3	18.6	18.5	18.1	17.6	17.2
	60th	17.8	18.0	18.0	17.4	17.1	16.6
	50th	17.4	17.7	17.7	17.1	16.8	16.4
	40th	17.1	17.4	17.3	16.7	16.3	16.1
	25th	16.5	16.9	16.7	16.2	15.9	15.5
	20th	16.3	16.7	16.5	15.9	15.6	15.1
SMI, kg/m^2^	80th	7.86	7.99	7.93	7.70	7.52	7.11
	75th	7.74	7.86	7.85	7.58	7.42	7.02
	60th	7.46	7.60	7.54	7.26	7.04	6.88
	50th	7.30	7.45	7.40	7.08	6.94	6.75
	40th	7.13	7.32	7.24	6.92	6.75	6.57
	25th	6.84	7.06	6.98	6.64	6.43	6.29
	20th	6.71	6.96	6.88	6.55	6.24	5.85
ALM_BMI_	80th	0.932	0.963	0.937	0.906	0.879	0.884
	75th	0.914	0.944	0.916	0.887	0.859	0.862
	60th	0.865	0.896	0.871	0.834	0.816	0.822
	50th	0.833	0.867	0.840	0.813	0.795	0.782
	40th	0.810	0.838	0.815	0.783	0.763	0.726
	25th	0.763	0.799	0.768	0.742	0.723	0.676
	20th	0.745	0.781	0.753	0.729	0.708	0.659

BMI = body mass index: weight (kg)/height (m)^2^, FM = fat mass, FMI = fat mass index: FM (kg)/height (m)^2^, FFMI = fat-free mass index: FFM (kg)/height (m)^2^, SMI = skeletal muscle mass index: appendicular lean soft tissue mass (ALM) (kg)/height (m)^2^. ALM_BMI_ = ALM-to-BMI ratio: ALM (kg)/BMI (kg/m^2^).

**Table 5:** Quartiles and quintiles of body composition indices, by age group (women).

Body composition	Percentiles	Age group					
parameters		Overall	65–69	70–74	75–79	80–84	85^+^
BMI, kg/m^2^	80th	25.2	25.0	25.2	25.4	25.2	25.8
	75th	24.6	24.5	24.6	24.7	24.8	25.4
	60th	23.2	23.2	23.2	23.3	23.5	23.5
	50th	22.4	22.6	22.3	22.4	22.6	21.5
	40th	21.7	21.7	21.5	21.7	21.8	20.8
	25th	20.4	20.5	20.3	20.4	20.3	19.5
	20th	19.9	19.9	19.9	20.0	20.0	19.1
Percentage FM, %	80th	37.6	37.0	37.5	38.3	38.7	40.1
	75th	36.5	36.0	36.6	36.6	37.5	39.3
	60th	33.9	33.5	33.9	34.4	34.3	34.8
	50th	32.3	32.0	32.4	32.4	32.5	32.9
	40th	30.5	30.3	30.4	30.9	30.7	30.8
	25th	26.9	26.7	27.1	27.4	26.9	25.4
	20th	25.5	25.2	25.5	26.0	25.3	24.7
FMI, kg/m^2^	80th	9.39	9.19	9.33	9.58	9.84	10.39
	75th	8.91	8.67	8.88	9.12	9.24	9.65
	60th	7.81	7.77	7.81	7.87	8.06	7.88
	50th	7.25	7.27	7.15	7.30	7.27	7.18
	40th	6.58	6.49	6.55	6.75	6.60	6.33
	25th	5.58	5.54	5.54	5.64	5.64	4.98
	20th	5.11	5.08	5.14	5.27	5.07	4.68
FFMI, kg/m^2^	80th	16.2	16.3	16.2	16.3	16.1	15.8
	75th	16.0	16.2	16.0	16.0	15.9	15.7
	60th	15.5	15.7	15.4	15.5	15.4	15.3
	50th	15.3	15.4	15.2	15.2	15.2	15.1
	40th	15.0	15.1	15.0	14.9	15.0	14.7
	25th	14.5	14.6	14.5	14.5	14.5	14.3
	20th	14.3	14.4	14.3	14.2	14.2	13.7
SMI, kg/m^2^	80th	6.39	6.53	6.36	6.31	6.06	5.99
	75th	6.25	6.41	6.25	6.17	5.97	5.84
	60th	6.00	6.15	6.00	5.91	5.74	5.57
	50th	5.84	6.00	5.83	5.78	5.63	5.43
	40th	5.70	5.84	5.69	5.61	5.49	5.22
	25th	5.44	5.59	5.44	5.34	5.28	4.90
	20th	5.33	5.50	5.35	5.21	5.16	4.78
ALM_BMI_	80th	0.670	0.697	0.670	0.647	0.627	0.573
	75th	0.654	0.680	0.652	0.633	0.606	0.561
	60th	0.612	0.636	0.619	0.597	0.567	0.528
	50th	0.590	0.611	0.594	0.574	0.545	0.513
	40th	0.566	0.593	0.568	0.552	0.518	0.498
	25th	0.531	0.561	0.538	0.513	0.483	0.459
	20th	0.514	0.547	0.523	0.504	0.472	0.448

BMI = body mass index: weight (kg)/height (m)^2^, FM = fat mass, FMI = fat mass index: FM (kg)/height (m)^2^, FFMI = fat-free mass index: FFM (kg)/height (m)^2^, SMI = skeletal muscle mass index: appendicular lean soft tissue mass (ALM) (kg)/height (m)^2^. ALM_BMI_ = ALM-to-BMI ratio: ALM (kg)/BMI (kg/m^2^).

## Discussion

We used DSM-BIA to determine age- and sex-specific values and percentiles for body composition parameters considered to be important health indicators in older adults. Our study populations from four cohort studies were homogeneous. FFMI and SMI significantly decreased with age in both sexes, although the FFMI remained constant among the women with only a 1% decrease up to age 84 years. In contrast, FMI did not change in either sex. Consequently, only male BMI significantly decreased with advancing age. The present data can be used for comparative assessments of healthy older Japanese and for international comparisons. In addition, because we previously reported age- and sex-specific reference values and appraisal standards for physical performance measures of older Japanese [28], these previous and present data will be useful for future studies of sarcopenia.

Our study sample was fairly representative of the general older Japanese population. We compared BMIs of our participants with national BMI statistics (the National Health and Nutrition Survey [29]) between 2008 and 2012. The mean BMI of our participants aged 70 years or older (23.3 kg/m^2^ in men and 22.7 kg/m^2^ in women) was almost the same as that for the national BMI statistics between 2008 and 2012 (approximately 23.2 kg/m^2^ in men and 22.9 kg/m^2^ in women). The distribution of BMI categories (<18.5 kg/m^2^, 18.5–24.9 kg/m^2^, and ≥25 kg/m^2^) was also similar between our male participants (4.9%, 68.8%, and 26.3%, respectively) and national data for males (approximately 6.2%, 67.2%, and 26.6%, respectively), which suggests our male sample was typical of the older male Japanese population. In contrast, the percentage of our female participants with a BMI ≥25 kg/m^2^ (22.3%) was lower than that in the national data (approximately 26.6%), although the proportion of our female participants with a BMI <18.5 kg/m^2^ (8.4%) was similar to that in the national data (approximately 9.7%). This indicates that, among women, FM, percentage FM, and FMI in particular might be slightly underestimated in our sample. However, it is very challenging at present to acquire representative data on body composition as measured by DSM-BIA among older Japanese adults. Therefore, we believe that our results are the best determination of body composition reference values in older Japanese adults.

Yamada et al [30]. found an individual age-related decrease in SMI and its 20^th^ percentile as measured by DSM-BIA (InBody 720) in both sexes. They reported a 20^th^ percentile SMI for people aged 65–79 years that was slightly higher than our result. However, the authors of that study [30] noted that their participants might not be a representative sample because they were recruited by advertisements at fitness and community centers, which therefore limited study participation to visitors of those fitness and community centers. This difference in sampling method could explain the discrepancy in study results.

As compared with results from Western populations [15, 31–33], the present values for BMI, FM, FMI, FFM, FFMI, ALM, and SMI were substantially lower for the same ages in both sexes. The discrepancies are mainly due to differences in original height and weight between Western and Japanese populations. Nevertheless, the age-related decreases in FFM, FFMI, ALM, and SMI may be broadly shared. Both the present results and those from previous cross-sectional [14, 15] and longitudinal [34,35] studies of Western populations show that FFM was greater in men than in women and that age-related change was more pronounced in men. Moreover, a greater percentage change occurred in ALM and SMI than in FFM and FFMI in the present study. This was also consistent with a previous report suggesting that loss of FFM is largely due to skeletal muscle loss, while other organ tissues are likely preserved [32].

A noteworthy finding of the present study is that the extent of the age-related decrease in FFMI among women was smaller than that in other populations [15, 36]. The FFMI of Italian women in their 70s decreased by approximately 5.3% as compared with those in their 60s [15]. In Chinese women, FFMI decreased by approximately 3.7% among women in their 70s as compared with those in their 60s [36]. In contrast, the FFMI remained constant among the women in the present study, with only a 1% decrease up to age 84 years. Although the FFMI of Japanese women was lower than in other populations, this smaller age-related decrease in FFMI may be a noteworthy characteristic of body composition change in older Japanese women, who have the longest life expectancy in the world.

There are conflicting results from cross-sectional and longitudinal studies of age-related change in FM. Cross-sectional analyses in Western [15, 31] and Japanese [1, 37] populations found that the absolute FM of people in their 70s was lower than that of people in their 60s in both sexes. However, longitudinal analyses show that FM was nearly unchanged for both sexes until around 80 years of age [35, 38, 39] or increased during the seventh and eighth decades of life in men or both sexes [1, 34, 40]. Our results show that FM in men and women barely changed until age 79 years, which was similar to the results of longitudinal studies. Although a stable or increased FM with aging may also be a characteristic of older Japanese, future research should include people aged 80 years or older, because FM would be expected to decrease at some age (e.g., 80 years or older), as suggested by the present and several previous studies [13, 34].

Our results are strengthened by the large sample size resulting from combining data from four cohorts. The inclusion of data from people aged 80 years or older is also a strength because few previous reports have investigated this age group. In general, fewer men than women participate in population-based studies; however, the random recruitment of a large number of male participants enabled us to present reliable age- and sex-specific reference values for men. The prevalence of low SMI, based on AWGS criteria, and the 20^th^ percentiles of SMI and ALM_BMI_ presented in our study will be useful for future studies of sarcopenia.

Our study has some limitations. First, as mentioned earlier, selection bias is a possibility. Second, as compared with DXA, DSM-BIA likely underestimates LM and overestimates FM among older Japanese, although we confirmed that DSM-BIA had acceptable accuracy [10, 12]. We need to be mindful of systematic bias when considering the results of DSM-BIA. Third, we did not determine the hydration status of the participants before body composition assessment. Because BIA works well in healthy individuals with a stable water and electrolyte balance, our findings may not be applicable to people with lumbar hyperlordosis or those who cannot remain immobile, are extremely overweight, or have an abnormal hydration status. Fourth, we acknowledge that our cross-sectional results are only indirect evidence of age-related changes. Finally, we did not examine the clinical importance of the present reference values. However, we expect that future longitudinal studies will use these reference values to examine association of the body composition indices with adverse health outcomes.

## Conclusions

This pooled analysis yielded age- and sex-specific body composition reference values and percentiles in nondisabled, community-dwelling, older Japanese adults. The small age-related decrease in FFMI may be a noteworthy characteristic of body composition change in older Japanese women. The data presented in this study should be useful in the clinical evaluation of body composition of older Japanese and for international comparisons.

## Supporting Information

S1 Table Characteristics of male participants, by cohort. KUSATSU = Kusatsu Longitudinal Study, HATOYAMA = Hatoyama Cohort Study, ITABASHI = Itabashi Cohort Study, KASHIWA = Kashiwa Cohort Study, BMI = body mass index: weight (kg)/height (m)^2^, FM = fat mass, FMI = fat mass index: fat mass (kg)/height (m)^2^, FFM = fat-free mass, FFMI = fat-free mass index: fat-free mass (kg)/height (m)^2^, LSTM = lean soft tissue mass, SMI = skeletal muscle mass index: appendicular lean soft tissue mass (kg)/height (m)^2^. *Low SMI is defined as <7.0 kg/m^2^ using the Asian Working Group for Sarcopenia algorithm [4].
(XLSX)

S2 Table Characteristics of female participants, by cohort. KUSATSU = Kusatsu Longitudinal Study, HATOYAMA = Hatoyama Cohort Study, ITABASHI = Itabashi Cohort Study, KASHIWA = Kashiwa Cohort Study, BMI = body mass index: weight (kg)/height (m)^2^, FM = fat mass, FMI = fat mass index: fat mass (kg)/height (m)^2^, FFM = fat-free mass, FFMI = fat-free mass index: fat-free mass (kg)/height (m)^2^, LSTM = lean soft tissue mass, SMI = skeletal muscle mass index: appendicular lean soft tissue mass (kg)/height (m)^2^. *Low SMI is defined as <5.7 kg/m^2^ in women using the Asian Working Group for Sarcopenia algorithm [4].
(XLSX)

S3 Table Weighted means of body composition parameters obtained from random effects meta-analysis model, by sex and age group, across cohort studies. CI = confidence interval. All *P* values for Cochran's *Q* statistic exceed 0.05. BMI = body mass index: weight (kg)/height (m)^2^, FM = fat mass, FMI = fat mass index: fat mass (kg)/height (m)^2^, FFM = fat-free mass, FFMI = fat-free mass index: fat-free mass (kg)/height (m)^2^, LSTM = lean soft tissue mass, SMI = skeletal muscle mass index: appendicular lean soft tissue mass (kg)/height (m)^2^.
(XLSX)

S4 Table Quartiles and quintiles of weight, FM, FFM, and ALM, by sex and age group. FM = fat mass, FFM = fat-free mass, LSTM = lean soft tissue mass.
(XLSX)
